# To Dr. Bradley

**Published:** 1802-03

**Authors:** L. Maclean

**Affiliations:** Sudbury


THE
Medical and Phyfical Journal.

VOL. VII.] MARCH, 1802. [NO. XXXVII.

### To Dr. BRADLEY.

Dear Sir,

THE operation of Tapping during Pregnancy, has, I perceive, engaged the attention of fome of your ingenious Correfpondents. It is, doubtlefs, a fubject of confiderable intereft and importance, in fo far as the life, not only of the fœtus, but even of the mother, may depend on the greater or lefs fkill and dexterity with which it is performed. If a Cafe in which the water was drawn off from the cavity of the abdomen five times, from the fixth week of conception to within twenty-five days of delivery, without inconvenience either to the mother or fœtus, fhould be deemed worthy of a place in your valuable Journal, the following, which occurred fome years ago in my practice, is at your difpofal. Befide furnifhing an ample field for fpeculation to the ingenious Phyfiologift, it may poffibly ferve as a guide to the inexperienced practitioner, fhould fimilar inftances fall under his care. It is intended for publication as foon as my avocations will permit, with many other, I truft, interefting Cafes, in an Appendix to a work on Hydrothorax, which has been for fome time nearly ready for the prefs.

On the 13th of June, 1791, I was defired to vifit Mrs. C. of ——— Hall, in Effex, a married woman, in the 34th year of her age. I found her labouring under confirmed genuine afcites, unaccompanied with the flighteft traces of anafarca in any part of the body. She had in a certain degree the characteriftic emaciation of this difeafe, but not, its general concomitant, debility. The appetite, bodily ftrength, and indeed all the bodily functions, with an exception or two that fhall be prefently noticed, were unimpaired. The eye and countenance too, had loft none of their wonted animation, for fhe was remarkable for the vivacity and gaiety of her difpofition. The urine, fhe imagined, was fomewhat diminifhed in quantity, but natural in appearance. The origin of the dropfical affection was dated about two years back; and it was attributed to an injury fhe fancied fhe received in her laft and only lying-in, five years before: This opinion feemed countenanced by the catamenia having been more or lefs irregular, both as to time and quantity, ever fince. She was fuppofed to have inherited a fcrophulous diathefis, feveral of her family having died of what was pronounced *Phthisis Scrophulosa.*

Various remedies had been ufed without any advantage; fhe had been tapped three times; and the water was rapidly accumulating, though evacuated only four days before.

After attentively confidering all the circumftances of the Cafe, I had no hefitation in afcribing the dropfy to difeafe of the right ovarium: This I found to have been the opinion of Dr. Orme, of London, who was confulted in the early period of the diforder; and it gained additional confirmation from the facts communicated by Mr. Gretton, a refpectable practitioner of Colchefter, who had always attended her. He affured me, that on evacuating the water an evident fullnefs or tumefaction could be felt in the right fide, apparently in the region of the ovarium.

The common remedies were adminiftered in various combinations for fome weeks, but without any benefit. They could not, however, be faid to have had a fair trial, having been taken only in fmall quantity, and very irregularly, owing to a peculiar irritability of the ftomach to almoft all diuretic medicines. Though every precaution was taken by the addition of the moft grateful aromatics, and fmall dofes of opium, yet they were feldom retained long enough to act upon the habit. As I could not poffibly be aware of what was afcertained fome weeks afterwards, that my patient was pregnant, this peculiar ftate of the ftomach was at the time a fource of furprife and embarraffment to me, for the appetite and digeftion were good.

On my vifit, the 9th of Auguft, when the operation was judged neceffary, for the firft time fince my attendance, I found Mrs. C's mind ftrongly impreffed with the belief that fhe was pregnant. This fhe grounded on the abfence of the catamenia the two laft periods, and on her having the ufual fymptoms, efpecially ficknefs, and fometimes vomiting, in the morning.— After a lapfe of five years fince her lying-in, with every reafon to believe the exiftence of difeafe of one ovarium fo extenfive as to be the fole caufe of the dropfy, apparently extending its morbid influence to the uterus itfelf, (if a conftant irregularity in its functions ever fince were to be regarded as criteria to judge by) befides this organ having been furrounded by, or floating in, a body of water for more than two years, I could not be fuppofed to affent readily to her opinion. No inconvenience occurred either during or after the operation. When the water was evacuated, the hypogaftric region was fuller than ufual; fo much fo, as to induce me now to think her opinion well well founded. The enlarged ovarium was diftinctly felt on examination, but no tumour perceived externally.

In order to avoid repetition, the different times at which the operation was performed, and the quantity of water drawn off each time, are fpecified in the annexed Table, and only fuch circumftances mentioned as feemed worthy of being recorded.

Toward the middle of Auguft, every doubt as to her pregnancy was removed, for fhe quickened, the motion of the fœtus having been diftinctly and repeatedly felt by her.

On the 25th of September, tapping being again deemed expedient, additional precaution was neceffary, with a view of avoiding the hazard of abortion, or of wounding the diftended uterus, now emerged above the brim of the pelvis. I directed a cordial draught with twenty-five drops of laudanum to be adminiftered half an hour before the operation; and the patient being placed in the ufual pofture, I endeavoured to prefs the body of the uterus as far back as poffible with both my hands, at the place where the puncture was to be made, while, with my fingers, the integuments were at the fame time protruded forwards. By thefe precautions, the diftance between the parietes of the abdomen at this place was increafed, and the integuments rendered more tenfe. In the mean time, Mr. Gretton meafured on the trochar the length that was judged merely fufficient to enter the cavity of the abdomen; and by firmly placing the fore finger of the right hand as a guard upon it, prevented any more entering than was abfolutely neceffary. The puncture being then dexteroufly made, and the ftillette withdrawn, the water was evacuated more gradually, the mouth of the canula being now and then plugged up by a piece of wood prepared for the purpofe; and a more regular and uniform preffure was preferved by means of a proper bandage. No untoward circumftance occurred either during or after the operation.

Before the whole of the water was removed, the motion of the fœtus was not only felt with great force againft the hand, but diftinctly feen thro' the integuments and flannel bandage.

Nov. 9th. She had inceffant ficknefs and vomiting, with fevere uterine pains which threatened abortion, the whole of this morning. The breathing was much interrupted, and fhe fuffered great inconvenience from diftention of the integuments of the abdomen. As thefe evidently proceeded from inordinate preffure on the diaphragm, uterus, and other abdominal vifcera, the operation having been too long delayed, fo they all fpeedily vanifhed on the evacuation of the water. Some part of the fluid was tinged with blood, and confiderable delay and inconvenience were experienced from the preffure of the uterus

 againft against the canula; yet ſhe made no complaint either during or after the operation.

Dec. 30th. Bore the operation better than uſual, being in high ſpirits the whole time, though the motion of the fœtus occaſioned her much uneaſineſs, and though greater delay was experienced than before. During the latter months of pregnancy, ſhe became, as may naturally be imagined, extremely heavy and unwieldy, eſpecially for ſome days before the two laſt tappings; yet her ſtrength, appetite, and natural vivacity continued unimpaired to the laſt; and on the 24th of Jan. 1792, ſhe was delivered of a ſtrong, full-grown boy, after a very eaſy and quick labour, before Mr. G. her *accoucheur*, or myſelf, arrived. The boy is now living, and is remarkably healthy and ſtrong of his age.

Her recovery was unuſually rapid, though there were ſeveral pints of fluid in the cavity of the abdomen at the time of her delivery, and though it accumulated ſo quickly afterwards as to require being drawn off in eleven days.

It may be worthy of remark, that after this period 'till the ſudden change which preceded and occaſioned her death, ſhe affirmed, her general health appeared rather to improve than otherwiſe; a circumſtance to be wondered at, conſidering the immenſe quantity of matter that had been for a great length of time collected within the body, as will be ſeen in the Diſſection. It ſerves however, I think, to illuſtrate and confirm a fact in Pathology which is now generally admitted, that what is called well digeſted or concocted pus, whether it be ſhut up in a cavity, expoſed to the air, or carried to the habit through the medium of the abſorbents, is productive of no inconvenience either to the ſurfaces on which it reſts, or to the conſtitution at large: So aware is every judicious ſurgeon of this, that he cautiouſly avoids expoſing the tender and irritable granulations of healing wounds and ulcers by wiping their ſurfaces too clean. It ſeems at the ſame time to prove, what is I think likewiſe allowed, and what I have more than once obſerved, that the ovaria are endowed with comparatively leſs acute feeling than perhaps any other part of the body; for in the preſent inſtance the mechanical ſtimulus of diſtention occaſioned no uneaſineſs. What other part or organ would have ſuffered ſo ſerious an injury, and ſo great a degree of diſtention, without ſerious inconvenience both local and conſtitutional?

It is intereſting too, to obſerve the influence preſſure had on the accumulated fluids during the laſt month, as might naturally be expected, when the uterus was attaining its utmoſt extent of ſize, and when, conſequently, the preſſure on the numberleſs numberlefs exhalant and abforbent mouths opening into the cavity of the abdomen was the greateft, the effufion was proportionably retarded. This did not occur fo uniformly and regularly in the preceding months, for the obvious reafon that it muft have been influenced by various caufes and circumftances which did not now exift. But while the mechanical influence of preffure, mufcular and arterial action, in promoting abforption and lymphatic circulation, is to be admitted to a certain extent, yet I am far from affenting to the notion of fome modern Phyfiologifts, that they are the fole agents concerned in producing thefe effects. This opinion has, I perceive, been recently advanced by a very ingenious Correfpondent in Vol. vi. p. 457,* of the Medical and Phyfical Journal, but is not fupported by any evidence tending to eftablifh its validity. Among many arguments, and, I think, facts that might be adduced in proof of the contrary; viz. that the abforbents are endowed with active, living, mufcular powers, and that they *actively* abforb as well as tranfmit their refpective fluids, in a certain degree totally, independent of mechanical impulfe, I fhall only mention one, which appears to me to afford decifive evidence in its favour. By the ufe of Digitalis various cavities are often fpeedily unloaded of enormous quantities of ferous fluid, when there is pofitive proof of diminifhed, mufcular, arterial, and every other impulfe or action, in confequence of the powerfully fedative and debilitating effects of certain quantities of this herb; and this has happened fometimes from the cavity of the abdomen, where the greateft preffure that could be borne proved of no avail.

The operation was in this cafe performed in the ufual place on the left fide. Would a puncture with a lancet at the umbilicus, as recommended by the learned Prefident of the London Medical Society, Dr. Sims, be a more fimple, eafy, or fafe mode of drawing off the water in fimilar cafes? I am induced to think it would not. It might, probably, fucceed equally well in the early ftages of pregnancy; but the preffure of the uterus, after the fourth month, againft the opening thus made, would very foon be fuch as to require the introduction of a female catheter, or fimilar inftrument, to pufh it backwards; and it is well known, that a round inftrument paffed into an opening

* Thefe are the authors words, in a note fubjoined to his interefting communication; "The phrafe *lymphatic tranfmiffion*, is here fubftituted for the ordinary term *abforption*, it being my opinion that the lymphatic veffels do not in any inftance *actively abforb*, but *paffively admit* the redundant, exhalant, interftitial, or extravafated fluids forced into their *permanently open orifices* by arterial and mufcular impulfe."

opening made with a lancet would occafion much pain and inconvenience; more fo, I am perfuaded, than our patient experienced. For thefe reafons, the method adopted with fo much fuccefs in the Cafe of which the hiftory has been given, claims, I think, a decided preference. A large female catheter was occafionally introduced through the canula.

*Dates of the different Tappings, &c.*

| When tapped. 1790. First time. | No. of pints. | Days intervening. | Pounds weight. |
|---|---|---|---|
| Aug. 23. | 11 | — | 12½* |
| 1791. | | | |
| March 23. | 25 | 212 | 28½ |
| June 9. | 33 | 78 | 38 |
| Aug. 9. | 33 | 61 | 37 |
| Sept. 25. | 37 | 33 | 42 |
| Nov. 9. | 38 | 45 | 41 |
| Dec. 30. | 12½ | 51 | 14 |
| 1792. | | | |
| Jan. 24. Delivered. | | | |
| Feb. 4.† | 27 | 36 | 30½ |
| March 2. | 18½ | 26 | 20 |
| April 11. | 44 | 40 | 51 |
| May 9. | 44 | 28 | 49½ |
| June 6. ‡ | 36 | 28 | 40 |
| June 11. Died. | | | |
| | 8 ) 359 ( | | 404 |
| | Gallons, 44 : 7 Pints. | | |

DISSECTION.

Permiffion having fortunately been obtained to infpect the body, Mr. Gretton very obligingly attended to conduct the diffection. On opening the abdomen, a fac of confiderable fize was obferved in the right fide of the hypogaftrium, and nearly filling the right Iliac region. This was found to be the right ovarium attached to the uterus by a thick neck, and adhering anteriorly

* This was not weighed, only gueffed at; but all the other times the water was accurately weighed and meafured, either by, or in the prefence of the hufband of the lady; and from the period of my attendance, in my prefence alfo.

† About an ounce of thick good pus iffued from the opening, at the clofe of the operation.

‡ The evening after the operation of this date, a train of fymptoms came on, which left no room to doubt the gradual approaches of the fatal event which foon followed teriorly to fome extent to the peritoneum. It was partially collapfed, owing to about two pints of its contents, a thick, brown, well digefted pus, having efcaped through an opening on one fide, fufficiently large to admit three fingers into the cavity of the abdomen. Neither the uterus nor left ovarium fhewed any marks of difeafe. The os tincæ feemed to be clofed by a gelatinous like fubftance, which induced me to think a fecond impregnation had taken place. But my much efteemed friend and Preceptor, the celebrated Profeffor Monro, to whom the parts were tranfmitted, after having been carefully removed and preferved in fpirits, affured me that this was not the cafe.

On dividing the fac, it was very thick but of a foft and fpongy texture, extremely irregular on its internal furface, and containing upwards of three pints of matter fimilar to that found in the cavity of the abdomen. The external furface of the inteftines, both great and fmall, was very confiderably inflamed, verging in fome places on gangrene; and it was fmeared all over with the matter which efcaped from the abfcefs, of which the greater part was contained in the pelvis. The liver was fofter in its texture; and its connecting ligaments more relaxed and elongated than natural. The other vifcera, as well as thofe of the thorax, were found. The head was not opened.

The inflammation of the inteftines, not the mere rupture of the abfcefs, was, without doubt, the immediate caufe of death; and that this was occafioned by the morbid ftimulus of the purulent difcharge from the fac feems equally probable; which fhews, that though good pus be productive of no inconvenience to the furfaces by which it is fecreted, it is highly noxious to others.

Had this Cafe occurred to me at an earlier period, before fuppuration, and impregnation had taken place, and before the ftomach had, by fympathy with the uterus, in confequence of the latter, acquired fuch inordinate fufceptibility to medicine, I think it probable the event might have been more favourable. I am inclined to draw this inference from the fortunate refults of two fimilar Cafes which fell under my care not long afterwards.

Mrs. R. the fubject of the firft Cafe, was a married woman of forty-fix, the mother of a numerous progeny. She had confirmed afcites, without any anafarcous fwelling, of three years ftanding, evidently arifing from difeafed and confiderably enlarged ovarium. The abdomen was fo large and tenfe, and fhe fuffered fo much inconvenience, that I advifed tapping before I put her on a courfe of medicine. To this, however, fhe pofitively refufed her affent. She was completely cured, and is now in perfect health, by a combination of calomel, fquills, cryftals cryftals of tartar, other diuretics, and ultimately tonics, perfevered in for fome weeks. She firft confulted me in Auguft, 1792, and fhe has had no relapfe fince.

The fubject of the fecond, was a Mrs. B. the wife of the Rev. Mr. B. late of Bury. She had a confiderable enlargement of one ovarium for many months before any figns of dropfy appeared. For this Mr. Cline was confulted. He pronounced upon the Cafe, and prefcribed for it, with his ufual accuracy and judgement. When my attendance was firft defired, the 31ft of Auguft, 1792, the abdomen was confiderably diftended with water; and the leg and thigh correfponding with the difeafed ovarium were anafarcous to a high degree: This laft, indeed, began to appear for fome months before there was any ferous effufion in the abdomen. She was likewife perfectly cured by a fimilar plan; and before fhe left this country, about three years afterwards, had two children. I have not heard of her lately.

I am, &c.

*Sudbury, Feb. 6, 1802.* L. MACLEAN.

---

### To Dr. BRADLEY.

Dear Sir,

